# Increasing disparities in the proportions of active treatment and 5-year overall survival over time by age groups among older patients with gastric cancer in Korea

**DOI:** 10.3389/fpubh.2022.1030565

**Published:** 2023-01-09

**Authors:** Hyun-Soo Zhang, Dong-Woo Choi, Han Sang Kim, Hye Jung Kang, Hoyol Jhang, Wonjeong Jeong, Chung Mo Nam, Sohee Park

**Affiliations:** ^1^Department of Biostatistics, Graduate School of Public Health, Yonsei University, Seoul, Republic of Korea; ^2^Department of Biomedical Informatics, College of Medicine, Yonsei University, Seoul, Republic of Korea; ^3^Data Link and Operations Team, Cancer Big-Data Center, National Cancer Center, National Cancer Control Institute, Goyang, Republic of Korea; ^4^Division of Medical Oncology, Department of Internal Medicine, Yonsei Cancer Center, College of Medicine, Yonsei University, Seoul, Republic of Korea; ^5^Cancer Knowledge and Information Center, National Cancer Control Institute, National Cancer Center, Goyang, Republic of Korea; ^6^Department of Preventive Medicine, College of Medicine, Yonsei University, Seoul, Republic of Korea

**Keywords:** aged 85 and over, stomach neoplasms, therapeutics, survival, geriatric assessment (MeSH)

## Abstract

**Purpose:**

As older patients with gastric cancer increase in Korea, no consensus indicative of anti-cancer treatment exists for the oldest old (age 85+). We investigated potential disparities in the proportion of surgery-including active treatment and the degree of survival improvement over time by age groups, and whether heterogeneity exists in the protective effect of time period on overall survival (OS) by age at diagnosis clusters.

**Materials and methods:**

A nationwide cohort (*N* = 63,975) of older patients with gastric cancer (age at diagnosis 70+) in 2005–2012 were followed until the end of 2018. Patients were categorized into four time period groups by their year of diagnosis. Cancer treatment patterns and 5-year OS were analyzed accordingly, and a random coefficients Cox model with random intercepts and random slopes of time period by age at diagnosis clusters was employed.

**Results:**

The mean age of patients was 76.4, and 60.4% were males. Most patients had 0–1 comorbidities (73.3%) and low-risk frailty scores (74.2%). Roughly two-thirds of patients received some form of anti-cancer treatment (62.4%), and while the number of comorbidities and the proportion of high-risk frailty scores trended toward an increase, the proportion of patients receiving anti-cancer treatment increased from 58% in 2005–2006 to 69.6% in 2011–2012. The proportion of surgery-including active treatment increased to over 70% in the 70–74 years old group, while stagnating at 10% in the 90+ years old group. Differences in the slope of 5-year OS improvement resulted in a widening survival gap between the old (age 70–84) and the oldest old. The protective effect of time period on OS hazard in the oldest old was not monotonically reduced with increasing “chronological” age but varied quite randomly, especially among female patients.

**Conclusion:**

Our study showed no upper age limit in terms of benefiting from the advances in the detection and treatment of gastric cancer over time. Thus, “functional” age rather than “chronological” age should be the criterion for anti-cancer screening and treatment, and actual implementation of proven treatments in the oldest old patients to reduce their non-compliance with treatment in clinical practice is needed to improve gastric cancer survival for all.

## Introduction

The incidence of gastric cancer is notably high in Korea and other East Asian countries, approximately double the worldwide incidence rate (age-standardized rate 32.5 vs. 15.8 per 100,000) (1). Korea is also one of the fastest aging countries (2), and the increasing proportion of older (age 70+) patients with cancer is adding to Korea's cancer burden (3). Unsurprisingly, the number of cancer diagnoses is also rising in the “oldest old” (age 85+); however, no streamlined consensus exists for standardized treatment in this age group, such as the benefit of adjuvant chemotherapy (4–6). Korea's aging population, combined with its high incidence of gastric cancer, highlights the need for specialized care in older patients diagnosed with gastric cancer.

There have been positive improvements, such as earlier detection and better survival for patients with gastric cancer in Korea (7–9). However, disparities are known to exist by age groups within the cancer control continuum of detection, treatment, and survival (10, 11). We continue to underutilize geriatric assessments for personalized treatment strategies (12, 13), and older patients are diagnosed at more advanced stages (6), do not receive any treatment (14), and show poorer survival (5, 6). Hence, we need to further study the effect of age on the survival benefit of older patients with cancer (15), and specifically focus on the oldest old in this endeavor.

In order to explore potential disparities in the cancer control continuum over time by age groups among older patients with gastric cancer in Korea, we first examined the trends in gastric cancer stage compositions, active treatment proportions including surgery, and 5-year survival proportions by 5-year age groups and gender. We then investigated potential heterogeneity by chronological age at diagnosis (Dx) clusters in the effect of “time period (elapsed calendar time)” on overall survival (OS) in the oldest old patients with gastric cancer by gender, using a random coefficients model and adjusting for other important prognostic factors such as cancer treatment patterns, comorbidities, and frailties.

## Materials and methods

### Dataset and study design

Korea's national health insurance services (NHIS) is a universal single-insurer, overseeing insurance claims and reimbursements for all Korean citizens. Thus, the NHIS database includes exhaustive medical utilization and prescription records at the population-level (16). After masking personal information, the NHIS database was utilized to retrospectively examine all older patients with gastric cancer in Korea who were aged 70+ at the time of Dx. The study protocol was approved by the institutional review board of the Yonsei University Healthcare System (IRB 4-2021-0374), and informed consent was waived due to the study's retrospective access to secured, population-based data.

This study also utilized the Korea Central Cancer Registry (KCCR) to obtain the Surveillance, Epidemiology, and End Results (SEER) summary stage (17) of incident gastric cancers in Korea from the same time period (2005–2012). Although a standalone data source, the KCCR provided us with cancer stage by time period and age groups, which is not contained in the NHIS database due to its claims-based nature.

### Study population

Among the 65,708 patients (age at Dx 70+) diagnosed with gastric cancer from 2005 to 2012, 42 with negative survival times (i.e., inaccurate date-of-deaths) and 1,691 without health insurance payment information were excluded, resulting in a study population of 63,975 patients. Gastric cancer Dx was operationally defined as having an International Classification of Diseases, 10th edition code of C16.x as the main symptom with concurrent inpatient admission, and the date of gastric cancer Dx was defined as the first inpatient admission date (16).

### Outcome and variable definitions

The primary outcome of interest was overall survival (OS). Newly diagnosed older patients with gastric cancer from 2005 to 2012 were accrued, and survival status was followed up until the end of 2018, ensuring at least 6 years of follow up for all patients. The whole accrual period of 8 years (2005–2012) was divided into four 2-year periods to examine trends in cancer stage, active treatment, and 5-year survival proportions over time. A “time period (elapsed calendar time)” variable was defined with four levels corresponding to these four 2-year periods (Table 1).

**Table 1:** Baseline characteristics of the study population by the four time periods of gastric cancer diagnosis.

**Variables**	**Total (*N* = 63,975)**	**Year of gastric cancer diagnosis (Dx)**				
		**2005~2006 (*N* = 13,198)**	**2007~2008 (*N* = 15,436)**	**2009~2010 (*N* = 17,062)**	**2011~2012 (*N* = 18,279)**	***P*-value^a^**
**Age at Dx, mean (SD)**	76.40 (5.25)	76.21 (5.09)	76.43 (5.21)	76.42 (5.27)	76.60 (5.37)	<0.001
**Gender**, ***N*** **(%)**						0.007
Male	38,662 (60.4)	7,845 (59.4)	9,275 (60.1)	10,335 (60.6)	11,207 (61.3)	
Female	25,313 (39.6)	5,353 (40.6)	6,161 (39.9)	6,727 (39.4)	7,072 (38.7)	
**Income level (among 0**~**20)**, ***N*** **(%)**						<0.001
<6	17,246 (27.0)	3,658 (27.7)	4,295 (27.8)	4,565 (26.8)	4,728 (25.9)	
6~11	11,366 (17.8)	2,476 (18.8)	2,699 (17.5)	2,967 (17.4)	3,224 (17.6)	
12~16	1,6838 (26.3)	3,429 (26.0)	4,041 (26.2)	4,537 (26.6)	4,831 (26.4)	
≥ 17	18,525 (29.0)	3,635 (27.5)	4,401 (28.5)	4,993 (29.3)	5,496 (30.1)	
**Residential region**, ***N*** **(%)**						<0.001
Metropolitan	23,637 (36.9)	4,597 (34.8)	5,531 (35.8)	6,466 (37.9)	7,043 (38.5)	
Urban	27,111 (42.4)	5,508 (41.7)	6,577 (42.6)	7,217 (42.3)	7,809 (42.7)	
Rural	13,227 (20.7)	3,093 (23.4)	3,328 (21.6)	3,379 (19.8)	3,427 (18.7)	
**Comorbidities**, ***N*** **(%)**						<0.001
0~1	46,874 (73.3)	10,782 (81.7)	11,437 (74.1)	12,061 (70.7)	12,594 (68.9)	
2~3	14,381 (22.5)	2,140 (16.2)	3,368 (21.8)	4,187 (24.5)	4,686 (25.6)	
4+	2,720 (4.3)	276 (2.1)	631 (4.1)	814 (4.8)	999 (5.5)	
**Frailty score**, ***N*** **(%)**						<0.001
Low risk (<5)	47,491 (74.2)	10,695 (81.0)	11,804 (76.5)	12,363 (72.5)	12,629 (69.1)	
Intermediate risk (5~15)	15,280 (23.9)	2,392 (18.1)	3,393 (22.0)	4,340 (25.4)	5,155 (28.2)	
High risk (≥ 15)	1,204 (1.9)	111 (0.8)	239 (1.5)	359 (2.1)	495 (2.7)	
**Treatment pattern**, ***N*** **(%)**						<0.001
Non-compliance with treatment	24,031 (37.6)	5,544 (42.0)	6,164 (39.9)	6,772 (39.7)	5,551 (30.4)	
Chemo/Radiation only	7,495 (11.7)	1,327 (10.1)	2,036 (13.2)	2,109 (12.4)	2,023 (11.1)	
Surgery with pre/post Chemo/Radiation	5,416 (8.5)	1,140 (8.6)	1,378 (8.9)	1,379 (8.1)	1,519 (8.3)	
Surgery only	27,033 (42.3)	5,187 (39.3)	5,858 (38.0)	6,802 (39.9)	9,186 (50.3)	

Dx, diagnosis; SD, standard deviation.

a *P*-values for trend were calculated using linear regression for the continuous variable “age at Dx” and the Mantel-Haenszel chi-square test for categorical variables.

Income level, residential region, comorbidities, frailty score, and treatment pattern were set as additional independent variables. Income level was defined as a quartile grouping of a 20-level insurance payment variable, where the insurance payment level is determined by monthly average wages or household wealth (property and other owned goods). Residential region was grouped as either metropolitan (the 7 major cities of Korea), urban, or rural. The Charlson comorbidity index (CCI) was referenced to define the number of comorbidities from 1-year before gastric cancer Dx to the date of Dx, and was grouped as having 0–1, 2–3, or 4+ comorbidities. The “Hospital Frailty Risk Score” developed by Gilbert et al. (18) was used to calculate a patient's continuous frailty score from 1 year before gastric cancer Dx to the date of Dx, and was categorized into low (<5), intermediate (5–15), or high risk (>15) patients (18). Gastric cancer treatment patterns from the date of gastric cancer Dx to 1-year after Dx were grouped into one of the following four categories (10, 19) surgery only, surgery with pre- or post-operative chemotherapy and/or radiation therapy, systemic (chemotherapy and/or radiation) therapy only, or non-compliance with treatment (NCT; no surgery, chemotherapy, or radiation therapy records).

### Statistical analysis

**Figure 1:**
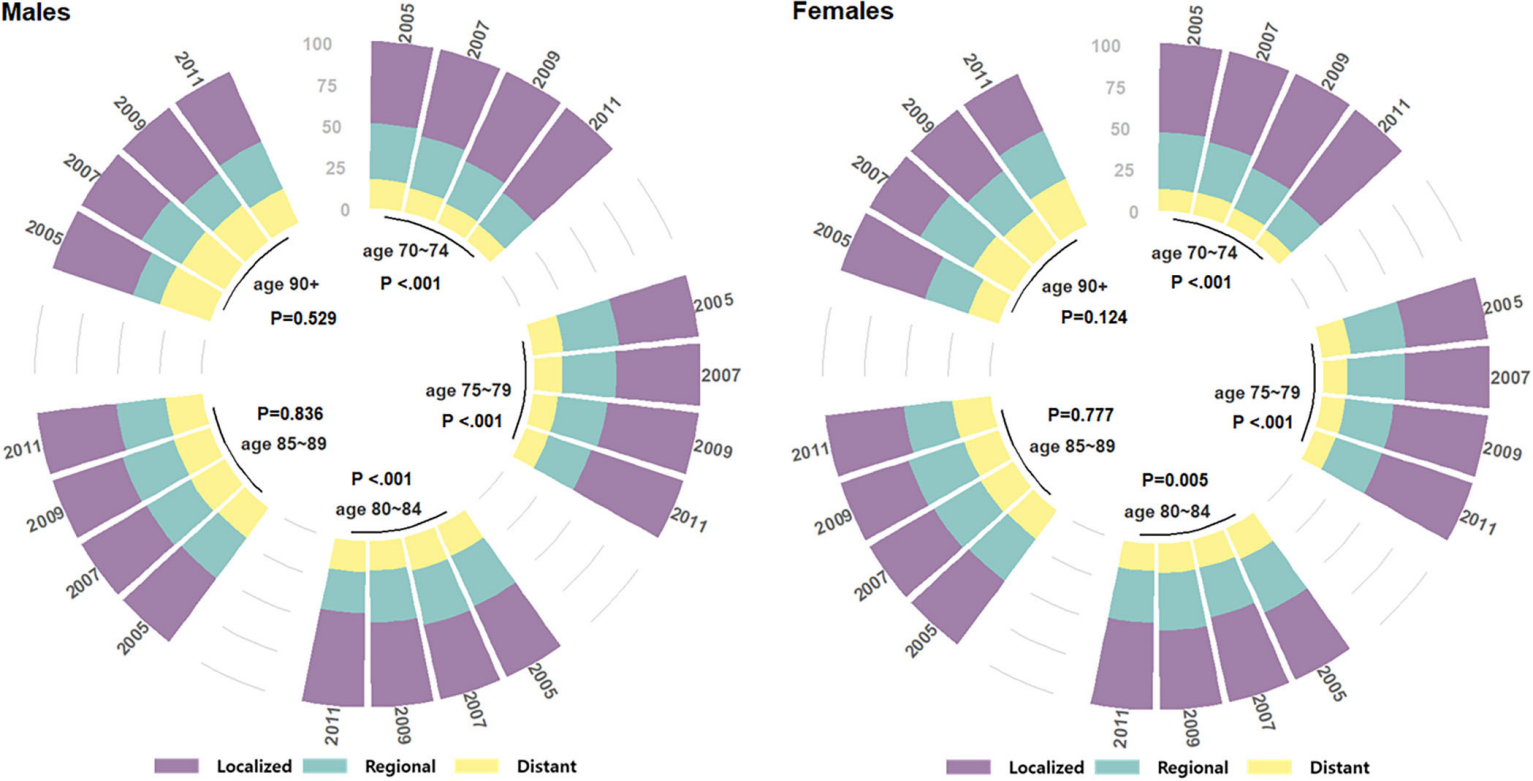
SEER stage distributions over the four time periods of gastric cancer diagnosis in 2005–2012, among older (age at Dx 70+) patients with gastric cancer in Korea.

A third-party data source (KCCR) was utilized to examine the SEER summary stage (17) distribution of older patients with gastric cancer in Korea from 2005 to 2012 by 5-year age groups and by gender (Figure 1). The SEER staging system is a combination of clinical and pathological documentation of the extent of disease, and its three summary stages are defined as (1) localized: malignancies confined to the organ of origin, (2) regional: cancer spread by direct extension to adjacent organs or tissues and/or spread to lymph nodes considered regional to the organ of origin, and (3) distant: cancer spread beyond adjacent organs or tissues and/or metastasis to distant lymph nodes. Although the cancer stage information pertained to the same patients diagnosed in the same time period as those of the current study population, this was standalone data not linked to our study population in the NHIS database. The Mantel-Haenszel chi-square test was used to test for a linear trend in cancer stage composition, proportion of active treatment including surgery, and proportion of 5-year survival over time in each age group by gender.

Statistical inference on OS was conducted by random coefficients Cox regression with a focus on possible heterogeneity by the age at Dx clusters (20, 21). Time to the outcome of interest (OS) was calculated as the time from Dx to all-cause death if deceased, or until the end-of-study (12/31/2018) if alive and right-censored. The regression model couldn't be explicitly adjusted for disease stage since it was accessed from a separate database (KCCR). The SEER summary stage changes over “time period” were statistically non-significant in the 85 to 90 and 90+ age groups in both genders (Figure 1), and therefore, only those whose age at Dx was 85+ were included in the regression model (male patients *N* = 2,522, female patients *N* = 3,105). Since the disease stage distribution of those included in the model didn't change over the time period of 2005–2012, disease stage was considered to be adjusted for when assessing time period's association with OS hazard. To investigate whether the effect of time period on OS hazard differed by a patient's age at Dx, random coefficients were employed by setting random intercepts by age at Dx clusters and random slopes for time period by age at Dx clusters (22). That is, the potential association of elapsed calendar time with OS hazard, in 2-year units over the four time periods, was allowed to vary randomly across age at Dx clusters (the random slopes of time period), as well as a patient's baseline frailty according to one's age at Dx (the random intercepts) (20, 21). The proportional hazards assumption for the main variable of interest “time period” was checked (Supplementary Table 1). Other independent variables were selected based on their prognostic importance in older patients with cancer (10, 23) and included in the model as possible confounders (Table 2).

**Table 2:** Random coefficients Cox regression with random intercepts and random slopes by age at diagnosis clusters.

**Gender**	**Variables**	**Coef**	**Hazard Ratio**	**95% CI**	***P*-value**
**Males (*****N*** = **2,522)**	**Age at diagnosis**				
	*Random intercept variance*	*0.0075*	*-*	*-*	*-*
	**Income level (0**~**20)**				
	<6 (Ref.)	0	1	-	-
	<12	0.021	1.02	0.9–1.16	0.75
	<17	0.026	1.03	0.92–1.15	0.66
	≥17	−0.012	0.99	0.89–1.09	0.82
	**Comorbidities**				
	0~1 (Ref.)	0	1	-	-
	2~3	0.015	1.01	0.92–1.12	0.77
	4+	−0.048	0.95	0.75–1.21	0.68
	**Frailty score**				
	<5 (Ref.)	0	1	-	-
	<15	0.0072	1.01	0.92–1.10	0.88
	≥15	0.21	1.23	0.98–1.56	0.093
	**Treatment pattern**				
	Non-compliance with treatment (Ref.)	0	1	-	-
	Chemo/Radiation only	0.24	1.27	1.13–1.43	<0.001
	Surgery with pre/post Chemo/Radiation	−0.33	0.72	0.54–0.96	0.028
	Surgery only	−1.018	0.36	0.32–0.41	<0.001
	**Time period**				
	1 unit increase	−0.045	0.96	0.92–0.99	0.021
	*Random slope variance*	*0.000074*	*-*	*-*	*-*
**Females (*****N*** = **3,105)**	**Age at diagnosis**				
	*Random intercept variance*	*0.035*	*-*	*-*	*-*
	**Income level (0**~**20)**				
	<6 (Ref.)	0	1	-	-
	<12	0.041	1.04	0.94–1.16	0.46
	<17	−0.032	0.97	0.88–1.07	0.52
	≥17	0.0042	1.00	0.92–1.10	0.93
	**Comorbidities**				
	0~1 (Ref.)	0	1	-	-
	2~3	−0.044	0.96	0.87–1.05	0.35
	4+	−0.0058	0.99	0.80–1.23	0.96
	**Frailty score**				
	<5 (Ref.)	0	1	-	-
	<15	−0.041	0.96	0.88–1.04	0.34
	≥15	0.060	1.06	0.87–1.29	0.55
	**Treatment pattern**				
	Non-compliance with treatment (Ref.)	0	1	-	-
	Chemo/Radiation only	0.18	1.20	1.08–1.33	<0.001
	Surgery with pre/post Chemo/Radiation	−0.80	0.45	0.32–0.64	<0.001
	Surgery only	−1.03	0.36	0.31–0.41	<0.001
	**Time period**				
	1 unit increase	−0.055	0.95	0.91–0.98	0.004
	*Random slope variance*	*0.00048*	*-*	*-*	*-*

Coef, regression coefficient; CI, confidence interval; Ref., reference group. Italics in the table values (random intercept variance, random slope variance) refer to “random” effects by each age at diagnosis cluster, by gender.

All tests performed were two sided, with statistical significance defined as *P* < 0.05. No missing values were present in the analyses after applying the study exclusion criteria. SAS 9.4 (Cary, NC, USA) and R 4.0.4 (The R foundation for Statistical Computing) were used for the analyses.

## Results

### Patient characteristics

**Figure 2:**
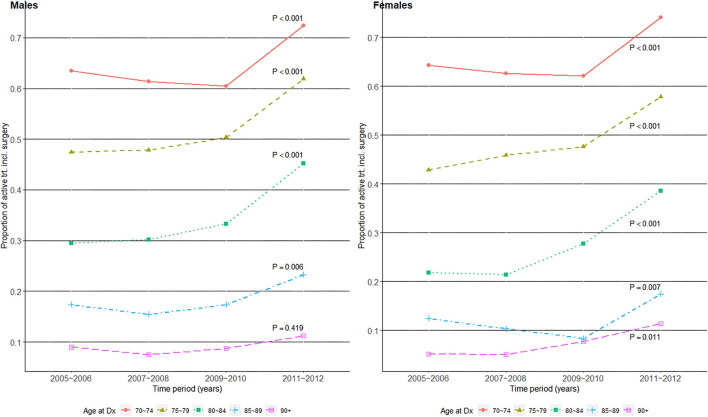
Surgery-including active treatment proportions over the four time periods of gastric cancer diagnosis in 2005–2012, among older (age at Dx 70+) patients with gastric cancer in Korea.

**Figure 3:**
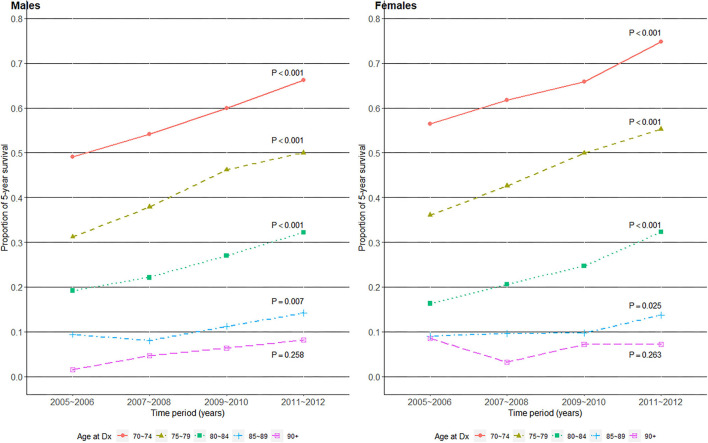
Five-year survival proportions over the four time periods of gastric cancer diagnosis in 2005–2012, among older (age at Dx 70+) patients with gastric cancer in Korea.

A total of 63,975 newly diagnosed gastric cancer patients with a mean follow-up of 4.52 years were analyzed in this study. Patient characteristics that include residential region, comorbidities, frailty score, and treatment patterns are summarized in Table 1. The average age of the study population was 76.4, with 60.4% being males. The majority of patients (79.3%) resided in either metropolitan (36.9%) or urban (42.4%) areas. Most patients had 0–1 comorbidities (73.3%) and a low risk profile for frailty (74.2%). Regarding treatment patterns, approximately two-thirds received anti-cancer treatment (62.4%), compared to NCT patients (37.6%). Although the number of comorbidities and the proportion of high risk frailty trended toward an increase over time, those who received anti-cancer treatment increased from 58% (year of diagnosis 2005–2006) to 69.6% (year of diagnosis 2011–2012). Characteristics such as age group composition, frailty, and treatment patterns differed by gender (*P* < 0.0001, 0.021 and <0.0001, respectively), as well as OS by gender (*P*-value 0.003), in favor of female patients. This is in agreement with the various differences by gender among patients with gastric cancer in Korea (7), and further analyses in the current study were thus conducted separately by gender (Figures 1–**3**, Table 2).

Next, gastric cancer stage distributions according to the SEER extent of disease (localized, regional, or distant) over the four time periods by age group and gender are shown in Figure 1. Statistically significant increases in localized disease were seen over time in the “old” age groups (70–74, 75–79, and 80–84) with *P* < 0.001, <0.001, <0.001 in males, and <0.001, <0.001, 0.005 in females, respectively. The “oldest old” age groups (85–89 and 90+) showed no significant change in stage composition over time, with *P*-values 0.836, 0.529 in males, and 0.777, 0.124 in females, respectively.

### Changes in active treatment proportions over time

We examined whether the proportion of patients who received surgery-including active treatment changed over time by age group and gender in Figure 2. Overall, the increase in active treatment proportion over time was statistically significant in all age groups in both genders except for male patients aged 90+, whose proportion of active treatment remained consistently around 10%. As the active treatment proportion increased to over 70% in the 70–74 years old group, it stagnated at 10% in the 90+ years old group in both genders, and their difference in active treatment proportion widened over time.

### Improvements in 5-year overall survival proportions over time

Next, we evaluated whether 5-year OS (the proportion of patients whose time to the outcome of interest (OS) ≥ 5 years) improved over time by age group and gender in Figure 3. Interestingly, all age groups in both genders, except those aged 90+, showed statistically significant improvements in 5-year OS proportions. The slope of increase was relatively flat in the 85–90 age group, compared to those of less old age groups in both genders. Differences in the slope of increase by age groups resulted in an increasing survival gap over time, especially between the old and oldest old.

### Random coefficients Cox regression of OS hazard upon time period

**Figure 4:**
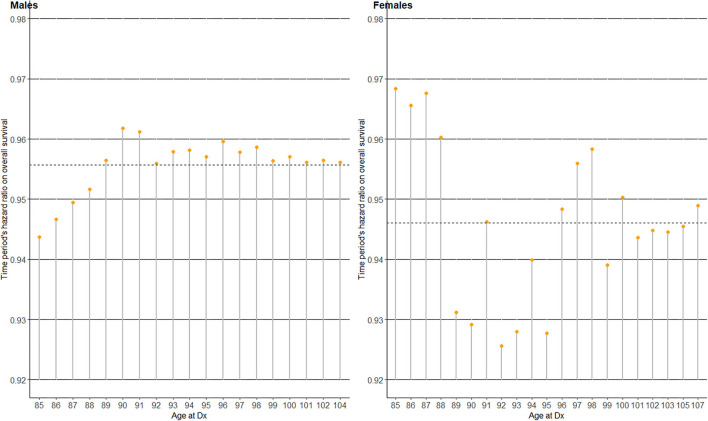
Time period's hazard ratio (HR) on overall survival (OS) for the four time periods in 2005–2012, among the oldest old (age at Dx 85+) patients with gastric cancer in Korea.

To investigate whether the OS benefit over time varied by patients' age at Dx clusters, random coefficients Cox regression utilizing the previously defined time to the outcome of interest (OS) was conducted in the oldest old by gender (Table 2 and Figure 4). The analysis included only the oldest old (age 85+) since their disease stage distribution was not statistically significantly different over time (Figure 1), and time period could thus be considered as being adjusted for disease stage in this sub-population of patients. The Cox regression coefficients (Coef) and resulting hazard ratios (HR) in Table 2 are all “fixed” effects, except the random intercept variance of age at Dx and the random slope variance of ‘time period’, which are “random” effects by each age at Dx cluster. Model comparison with and without the random effects (intercept and slope) *via* a chi-squared test showed a statistically significantly better fit for the model with the random effects in both genders (*P*-values 0.018 and <0.001 in males and females, respectively). The effect of elapsed calendar time on OS hazard over the four time periods was protective, i.e., OS of the oldest old patients with gastric cancer improved over the four time periods. The average HR of “time period” among those aged 85+ was 0.96 in males and 0.95 in females per one unit increase in “time period,” corresponding to two calendar years. Considering the variance of the random slope for “time period,” 95% of the age at Dx clusters had a time period HR within the interval $\exp\left(-0.045\pm 1.96\times \sqrt{0.000074}\right)=\left(0.94,\;0.97\right)$ in males, and $\exp\left(-0.055\pm 1.96\times \sqrt{0.00048}\right)=\left(0.91,\;0.99\right)$ in females (20). This leads us to Figure 4, which displays the best linear unbiased predictions (21) of time period's HR on OS by each age at Dx cluster by gender. Compared to the average “fixed” effect HR of time period (dotted horizontal line), male patients aged 85–88 at Dx showed a smaller HR and a larger protective effect of time period on OS hazard, while the HR of time period on OS varied quite randomly across age at Dx clusters in female patients.

## Discussion

This study showed that among older (age at Dx 70+) patients diagnosed with gastric cancer between 2005 and 2012 in Korea, cancer stage compositions, active treatment proportions including surgery, and 5-year survival proportions trended toward earlier detection with higher proportions of active treatment and 5-year survival in the old (age 70–84) group of patients, but not so in the oldest old (age 85+). We observed minimal changes in the detection and treatment of gastric cancer in the oldest old compared to those in the old, and the survival gap between the two patient groups increased over time. Among the oldest old, random coefficients Cox regression by age at Dx clusters showed no monotonically decreasing survival benefit of time period by increasing age, especially in female patients.

Korea's national cancer screening program, which advocates regular screening for various cancers in adults, has been a major factor in the earlier detection of gastric cancers. The uptake of gastric cancer screening increased from 48.5% in 2005 to 77.9% in 2012 across all ages, corresponding to an annual percentage change of 4.61% per year (24). Increased screening likely contributed to earlier detection at more “localized” stages (8) and subsequently to improvements in survival, with 5-year relative survival improving from 58% between 2001 and 2005 to 76.5% between 2013 and 2017 (7). The screening program's effectiveness was also demonstrated in a dose-response manner, where higher survival was observed by an increasing number of times screened (9). Our study's sub-population of old (age 70–84) patients showed similar findings, with a clear trend of increasing localized stages (Figure 1) and increasing 5-year survival proportions in both genders (Figure 3). However, the disease stage composition of the oldest old (age 85+) patients in both genders did not change toward earlier detection during the study period. This may be due to recommendations against screening in old ages (25), especially in the oldest old, due to potential harms outweighing the benefits of screening. Despite the potential harm perceived in the recommendations, routine screening with an endoscopy once every 2 years may be neither overly invasive nor emotionally stressful, and a recent report on the unexpectedly high rates of screening in adults aged 85+(25) may reflect their unmet needs of healthier, cancer-free aging.

The improvement in survival over time among older patients with gastric cancer may also be explained by the increasing proportion of patients who received active treatment that includes surgery. A first point of note is the abrupt increase in active treatment proportions in 2011–2012 compared with 2009–2010 in Figure 2. This coincides with the initiation of NHIS insurance coverage of endoscopic submucosal dissections (ESDs) from late 2011 (26), and the figure should thus be interpreted with caution as ESDs prior to 2011 were not captured. However, the overall trend of increasing active treatment proportions over time still reflects an earlier detection of surgically operable gastric cancers, and Choi et al. (26) also showed a shift in surgical treatment patterns from open gastrectomy to less invasive endoscopic or laparoscopic surgeries in Korea during this time span. Additionally, the NCT proportions decreased over time in our study, which agrees well with the trend of NCT among Korean patients with gastric cancer from a previous study (14). The previous study (14) also noted that the proportion of NCT increased with age, and that a large proportion of the older patients were NCT patients. The oldest old patients showed very low surgical treatment proportions of 10–20% (Figure 2) compared with their respective “localized” stage compositions of ~50% (Figure 1). As gastric cancer treatment with curative intent requires surgical resection, this indicates potentially suboptimal under-treatment in the oldest old population, although one's overall fitness or resilience to undertake cancer treatment should be evaluated in conjunction (12). Another study on the association of age with guideline-concordant cancer care also noted that old age itself was a predictive factor of NCT, regardless of patients' comorbidity or performance status (27). This supports our hypothesis that some patients in the current study's oldest old group may have been under-treated despite their ability to tolerate more aggressive treatment.

Shifting our focus to the 5-year survival proportions over time (Figure 3), a prominent increase was achieved in the 70–74 and 75–79 years old groups, especially among female patients. Although the slope of survival improvement relatively flattened, it was statistically significant even in the 85–89 years old group in both genders. While the 90+ years old group showed no such survival improvements, they had already exceeded Korea's average life expectancy of 83.4 years in males and 87.9 years in females (28) by the time of gastric cancer Dx. However, simply comparing one's age to the general population's life expectancy could be misleading, and a more refined approach such as the National Comprehensive Cancer Network guideline's life expectancy table by upper, middle, and lower quartiles of overall health status (29) may be more informative in oncological decision making. For example, even at the age of 90, the most robust males and females in the U.S. can expect 5.9 and 6.9 additional years of life, respectively. Accumulating evidence from clinical studies worldwide state that gastrectomy is feasible and effective among operable older patients (15, 23, 30, 31), and even that palliative chemotherapy is well-tolerated and prolongs survival in fit older patients with metastatic disease (5, 6). Therefore, communicating the available treatment options and the importance of a geriatric assessment's “functional” age (12, 13) over “chronological” age to these nonagenarian patients may empower their desired goals toward receiving more effective, individualized treatment.

To further ascertain the importance of functional age (12, 13) over chronological age, we investigated the survival benefit conferred over time separately by chronological age at Dx clusters amongst the oldest old group (Table 2 and Figure 4). Here, the survival benefit of “time period (elapsed calendar time)” refers to the fact that gastric cancer survival has improved steadily over time owing to earlier detection and better treatment options (7, 9, 26, 32), especially in Korea (Figures 1–3). Since the model with random effects by age at Dx clusters provided a statistically significantly better fit compared to one without, the oldest old patients with gastric cancer indeed showed heterogeneity in time period's protective effect on OS hazard by age at Dx clusters. However, time period's protective effect on OS hazard did not monotonically decrease by age such that the older the age, the smaller the survival benefit of time period (Figure 4). Although the survival benefit of time period decreased with increasing age in male patients aged 85–90, it reached a steady 4–5% reduction of OS hazard by time period in older ages. This decrease in survival benefit over time from ages 85–90 among male patients may be indicative of a hypothesized age-cutoff, where ages older than the cutoff are accompanied by survival hazards strong enough to offset the survival benefit of advances in gastric cancer detection and treatment accumulated over time. In contrast, the protective effect of time period varied quite randomly by each age at Dx cluster among female patients. That is, the survival benefit conferred over time varied regardless of increasing age, which strengthens the opinion that old age itself should not be a contraindication for applying anti-cancer treatment (5, 25, 29). Overall, our results provide further evidence to utilize functional age (12, 13) rather than chronological age in oncological decision-making, where in addition to geriatric assessments, more objective biomarkers of functional age are being actively pursued (4).

From a health equity perspective, while Korea experienced vast improvements in the detection, treatment, and survival of gastric cancers over the past two decades (7, 9, 26), cancer stage composition remained stagnant, the proportion of NCT remained high, and survival improvement was minimal in its oldest old subpopulation (Figures 1–3). This age-related difference in the degree of cancer survival improvement over time has been documented in the U.S. and Europe as well, where six of the seven cancers studied showed a widening survival gap between younger and older patients (11), and younger patients with gastric cancer experienced a stronger survival improvement than their older counterparts (10). To reverse this trend toward a survival benefit for all, conducting clinical trials to determine treatment response in older patients with cancer (11) and actual implementation of proven anti-cancer treatments in these patients should go hand in hand. This is especially true considering the current knowledge that at least some form of cancer treatment is better than none in terms of prolonging survival in fit older patients with gastric cancer (5, 6).

We also address some strengths and limitations of our study. First of all, we used a novel approach to examine whether a chronological age cutoff exists in terms of old age itself offsetting the survival benefit of time period accumulated through advances in the detection and treatment of gastric cancer over time. Additionally, we used nationwide data covering the entire population of older patients with gastric cancer in Korea, thus generating higher-level evidence. However, the absence of cancer staging information in the NHIS database is a limitation, for which we remedied as much as possible by separately obtaining SEER summary stage information from the KCCR. A second limitation is the absence of clinical variables such as lab tests, due to our data's claims-based nature. Third, although we were unable to fully adjust for a geriatric assessment's seven main domains (12), the CCI and frailty score were derived and included in the random coefficients analysis. Fourth, the proportional hazards assumption for time period 4 relative to time period 1 in the oldest old female patients may not be satisfied (Supplementary Table 1), and the results should thus be interpreted accordingly, as the hazards may not be constantly proportional over time in this situation.

In conclusion, we observed large and increasing disparities in the detection, treatment, and survival of gastric cancer among older patients in Korea, especially between the old (age 70–84) and oldest old (age 85+). These disparities essentially deprived the oldest old patients from reaping the survival benefits accumulated over time. However, the variation in the survival benefit conferred over time was not dependent upon “chronological” age, serving as further evidence to utilize “functional” age (12, 13) for oncological decision-making. To improve gastric cancer survival for all, we thus need to conduct more clinical trials based on functional age in the oldest old to target those who would benefit from anti-cancer treatment, and then actually implement those proven treatments in these patients to reduce NCT in clinical practice.

## Data availability statement

The datasets presented in this article are not readily available because the datasets accessed for this study are health insurance claims from the National Health Insurance Services (NHIS) of Korea, and due to privacy issues of individual health records, was granted access only through approval of the Yonsei University Healthcare System's Institutional Review Board (IRB). Requests to access the datasets should be directed to soheepark@yuhs.ac.

## Ethics statement

The studies involving human participants were reviewed and approved by Yonsei University Healthcare System, Institutional Review Board (IRB). Written informed consent for participation was not required for this study in accordance with the national legislation and the institutional requirements.

## Author contributions

H-SZ, SP, and CN: conception and design. H-SZ, D-WC, and SP: data collection. H-SZ, D-WC, HSK, HJK, HJ, WJ, and CN: analysis and interpretation of data. H-SZ, HSK, HJK, HJ, WJ, and SP: manuscript writing. H-SZ, D-WC, HSK, SP, and CN: revision of final article. All authors contributed to the article and approved the submitted version.
