# Supplementary material for: Increasing disparities in the proportions of active treatment and 5-year overall survival over time by age groups among older patients with gastric cancer in Korea
Source: Front Public Health. 2023 Jan 9;10:1030565. doi: 10.3389/fpubh.2022.1030565 (PMC9869046; doi:10.3389/fpubh.2022.1030565)
Supplement: Supplementary file 1 [file Table_1.docx]

Supplementary Material

# Supplementary Figures and Tables

## Supplementary Tables

Supplementary Table 1. Test of the proportional hazards assumption for overall survival (OS) hazard by time period (reference group: Time 1; 2005-2006) in each gender, among the oldest old (age at Dx 85+) patients with gastric cancer in Korea (2005-2012)

| **Supremum Test for Proportional Hazards Assumption** | | | | |
| --- | --- | --- | --- | --- |
| **Variable  (Category)** | **Males** | | **Females** | |
|  | **Replications** | **P-value** | **Replications** | **P-value** |
| Time 2; 2007-2008 | 1000 | 0.618 | 1000 | 0.150 |
| Time 3; 2009-2010 | 1000 | 0.212 | 1000 | 0.079 |
| Time 4; 2011-2012 | 1000 | 0.231 | 1000 | 0.001 |
